# Inadvertent Intrauterine Instillation of Trichloroacetic Acid During Sonohysterography

**DOI:** 10.7759/cureus.70825

**Published:** 2024-10-04

**Authors:** Alexandra Kuzma, Khooshali Shah, Caleb Kallen, Erin Jun

**Affiliations:** 1 Obstetrics and Gynecology, Crozer-Chester Medical Center, Upland, USA; 2 Obstetrics and Gynecology, University Hospitals Cleveland Medical Center, Willoughby, USA; 3 Reproductive Endocrinology and Infertility, Shady Grove Fertility, Philadelphia, USA

**Keywords:** fertility, in vitro fertilization ivf, medical error, trichloroacetic acid, uterovaginal chemical burn

## Abstract

Trichloroacetic acid (TCAA) solution is used to treat vulvar condyloma and cervical intraepithelial neoplasia. Saline infusion sonohysterography (SIS) detects intrauterine pathology and fallopian tube patency in infertile patients. A 33-year-old Caucasian nulliparous fertility patient presented to the emergency department with extensive burns following accidental intrauterine instillation of TCAA during SIS with tubal perfusion. On presentation, the patient was hemodynamically stable. There was extensive erythema and tenderness in a spill/burn pattern on both thighs as well as the groin, perineum, and labia. Speculum exam revealed white, leathery mucosa with the absence of normal vaginal rugae. The abdominal exam was notable for generalized tenderness without guarding or rebound. Complete blood count (CBC), serum electrolytes, and CT scan were unremarkable. The patient was admitted for observation and pain management. She received serial abdominal exams, topical aquaphor, vaginal estrogen, and serial labs and pelvic imaging. The patient remained stable and was discharged on hospital day two. At three months, the patient reported improvement in pain and gradual skin healing. She resumed fertility treatment at a different fertility center, and a frozen embryo transfer resulted in a live birth.

This case demonstrates severe vaginal and perineal injury after intrauterine instillation of TCAA. At three months, vaginal and external genitalia required ongoing burn care. Notably, endometrial recovery is evidenced by the delivery of a live birth after embryo transfer. The case underscores a preventable medical accident and highlights the regenerative ability of endometrial stem cells to respond to hormonal cues and restore endometrial receptivity after chemical trauma.

## Introduction

We report the evaluation and management of a patient after accidental intrauterine instillation of trichloroacetic acid (TCAA) during saline infusion sonohysterography (SIS) with associated tubal perfusion procedure. It denatures proteins, lyses cells, and produces chemical cauterization. Used topically in gynecology offices, TCAA treats condyloma acuminata and cervical intraepithelial neoplasia (CIN) [1-3]. When treating condyloma acuminata, TCAA is applied sparingly to the affected areas once per week for eight to 10 weeks [2]. When applied to areas of cervical dysplasia (CIN 1-3), TCAA produces high rates of CIN regression and remission and human papillomavirus (HPV) clearance [3]. In dermatology practices, TCAA produces a chemical peel for the treatment of acne scars [4]. Lastly, TCAA has been used for chemical endometrial ablation in women with abnormal uterine bleeding. A pilot, prospective randomized clinical trial to assess the safety and efficacy of TCAA found that intrauterine instillation of small volumes of TCAA resulted in reduced menstrual bleeding [5].

Trichloroacetic acid exposure can damage skin and adjacent structures with which the acid comes into contact. Symptoms of TCAA skin exposure include skin irritation, burning, pain, swelling tenderness, and skin ulcerations [6,7]. Accidents with TCAA present risks to patients and providers. In 2015, a gynecologist spilled 20 ml of 80% TCAA onto her right thigh and incurred second-degree burns as a result [6]. Despite the widespread use of TCAA in diverse clinical settings, reports of adverse events are rare, and management of excessive or accidental exposures is guided by the first aid principles of any toxic exposure.

Saline infusion sonohysterography is an office-based imaging study that is useful for detecting uterine pathology, including leiomyomata, polyps, adhesions, and anomalies (e.g., uterine septum). Saline instilled through a transcervical catheter distends the uterine cavity while transvaginal sonography is performed. In the past 15 years, several products have been developed to generate air contrast (bubbles) within the saline, which permits real-time evaluation of the fallopian tubes with ultrasound, dubbed 'saline tubal perfusion.' The observation of fluid bubbles traveling through the fallopian tubes and accumulating in the pelvic cavity indicates that one or both fallopian tubes are patent [8,9].

Here, we report a planned SIS-tubal perfusion study in an infertile patient who instead experienced instillation of TCAA into her uterus, fallopian tubes, and abdominal cavity, with reflux into the vagina and then spilling onto her perineum, thighs, and buttocks. The patient thus suffered from both internal and external TCAA burns, and our case study details her inpatient management in a community hospital setting. We highlight the importance of office-based protocols to protect against improper toxic exposures, and we describe the clinical course of this patient. We emphasize the value of a multidisciplinary team approach and offer guidance on this rare but complex exposure.

## Case presentation

A 33-year-old nulliparous Caucasian patient without significant past medical or surgical history presented to our emergency department in a community hospital for the management of external and internal burns following accidental exposure to 85% TCAA in an outpatient reproductive endocrinology and infertility office during a planned SIS-tubal perfusion study. We were informed that the patient experienced immediate burning as the study was underway but was initially told that burning is normal and so the study continued. We were informed that the left fallopian tube was noted to be patent during this evaluation, with copious flow of TCAA bubbles on that side and spillage into the pelvic cavity. There was no certain perfusion of the right fallopian tube, suggesting proximal obstruction on that side.

As the study progressed, the patient reported abdominal pain and abdominal cramping followed by severe burning sensations in her vulva, groin, and upper thigh. With the study nearly complete, the patient expressed a desire that the study be aborted, and she went to the restroom to clean up. Upon returning to the exam room, the patient was noted to have discrete, well-demarcated patches of erythema and irritation on her bilateral inner thighs, groin, and labia majora and minor. The provider realized that the 85% TCAA was infused instead of saline. The provider estimated that approximately 15 ml of 85% TCAA was infused into the uterus. Copious vaginal and perineal irrigation with normal saline was initiated. The patient was transported via ambulance to our hospital for admission to our burn unit.

On presentation, the patient was stable and had normal vital signs. The patient reported abdominal cramping and burning of the affected areas (vagina and exposed skin areas). Intravenous morphine sulfate was administered in the emergency department, and the patient reported improvement in pain from extreme to moderate. The patient’s physical examination was notable for diffuse areas of erythema and tenderness in a spill/burn pattern present on both upper and inner thighs bilaterally that encompassed her groin, perineum, and labia. These lesions were consistent with second-degree burns. An internal speculum exam was attempted and required a copious amount of lubricant to place the speculum into the vagina. A speculum exam was notable for diffuse, thick, white, leathery-appearing mucosa with the absence of normal vaginal rugae. There was no active bleeding nor any discrete lacerations/excoriations. Abdominal examination was notable for generalized abdominal tenderness on deep palpation without rebound, rigidity, or guarding. Basic bloodwork and electrolyte panels were drawn: CBC, comprehensive metabolic panel (CMP), phosphorus, magnesium, and lactate levels were within normal limits. A stat CT scan with oral contrast revealed no significant bowel injury or pathology and the presence of fluid within the endometrial canal.

Given the patency of the left fallopian tube during the study, there was concern for a possible delayed presentation of bowel injury. The case was discussed extensively with multiple sub-specialties, including emergency medicine, obstetrics and gynecology, the burn team, general surgery, toxicology, and the poison control center. Due to the patient’s reassuring abdominal exam, normal lab studies, and imaging, the concern for intra-abdominal injury was low. We considered expectant management (consisting of inpatient observation with serial abdominal exams, labs, and vital signs) or diagnostic laparoscopy to assess the pelvic organs. Ultimately, using shared decision-making between the patient and the multi-disciplinary team, the decision was made for observation with serial abdominal exams with a low threshold for diagnostic laparoscopy if the patient’s clinical status acutely worsened. Thus, the patient was admitted for observation and pain management.

Burn specialists suggested topical Aquaphor four times per day for the second-degree burns present on the patient’s perineum and upper thighs. The burn team acknowledged parallels between the effects of pelvic radiation and chemical burns on the uterus and vagina and thus sought guidance from gynecologic oncology. The gynecology oncology team suggested vaginal estrogen to decrease scar formation, to which the patient was amenable. The lubricant was generously spread over the applicator tip to improve comfort during the placement of the 1.25 mg vaginal estrogen, and this treatment was well tolerated by the patient.

The patient received serial abdominal exams and had repeat lab work done that remained unremarkable. Throughout her admission, there were no signs of an acute abdominal process. Her pain progressively improved and was then controlled with ibuprofen and acetaminophen, absent any opiates. Repeat CBC and lactate levels were within normal limits. Given her overall stable and improving clinical status, she was discharged home on hospital day number two with the following treatment plan: nightly vaginal estrogen to assist with healing of the vaginal mucosa and to minimize the risk of contractures of the vaginal canal; also a short course of oxycodone for episodes of more significant perineal pain.

One week following discharge, the patient was seen by the burn specialists and consulted with behavioral health. She was instructed to continue using Aquaphor on all healing areas and to wear silver patches as often as possible. At that time, the patient reported feelings of anxiety and stress. The patient met with a member of the behavioral health team to discuss her traumatic injury, and she was provided additional community resources. She was also referred to a pelvic floor physical therapist to assist with new symptoms of dyspareunia and dyschezia. Three months following discharge, the patient reported having a withdrawal bleed after treatment with estrogen and then progesterone. This response indicated some measure of recovered/healthy endometrium. The patient’s pelvic area at that time remained very sensitive, and subsequent speculum exams were performed under anesthesia.

**Figure 1:**
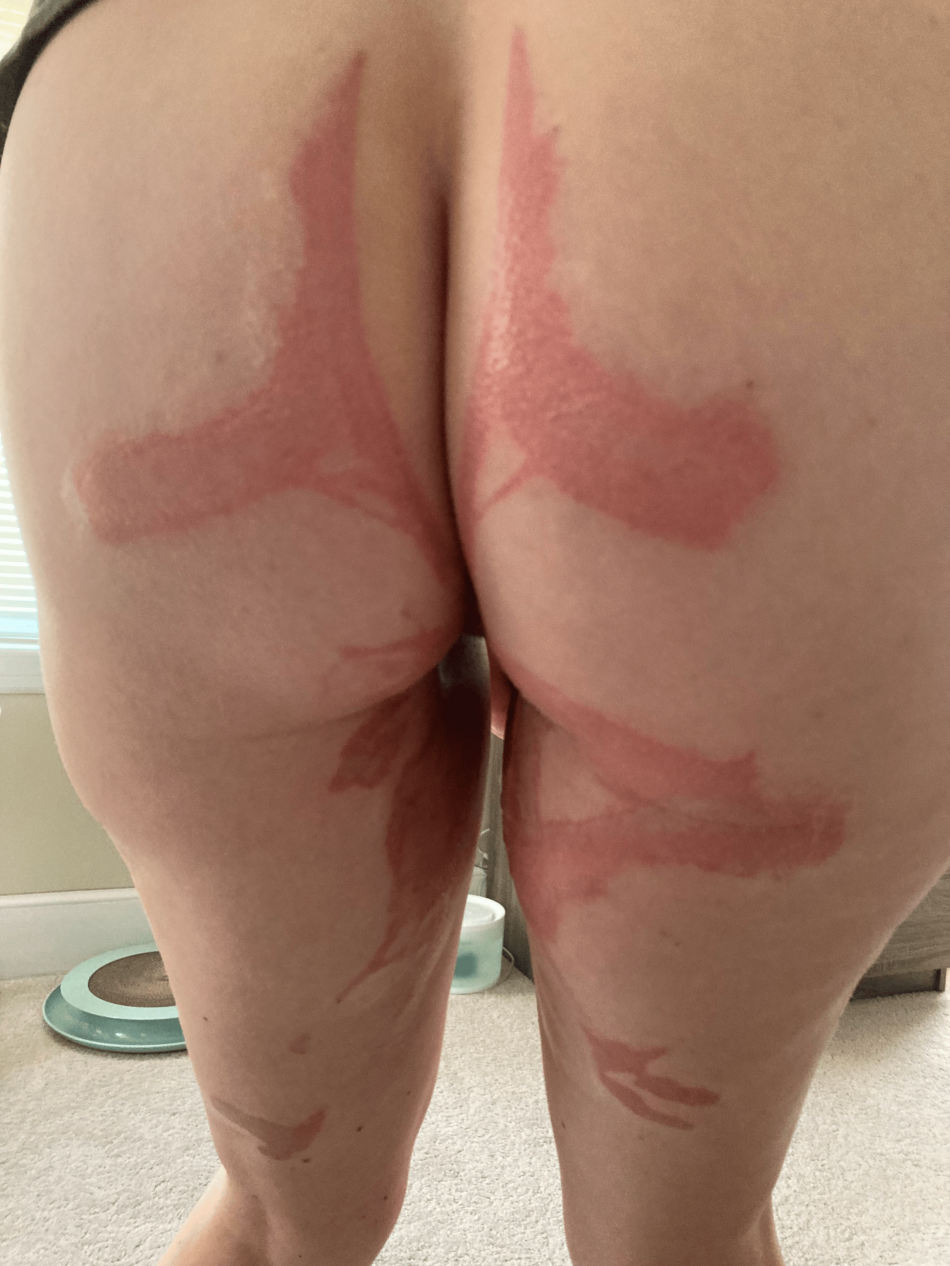
Skin burn patterns on the patient one year following the TCAA burn TCAA: Trichloroacetic acid

**Figure 2:**
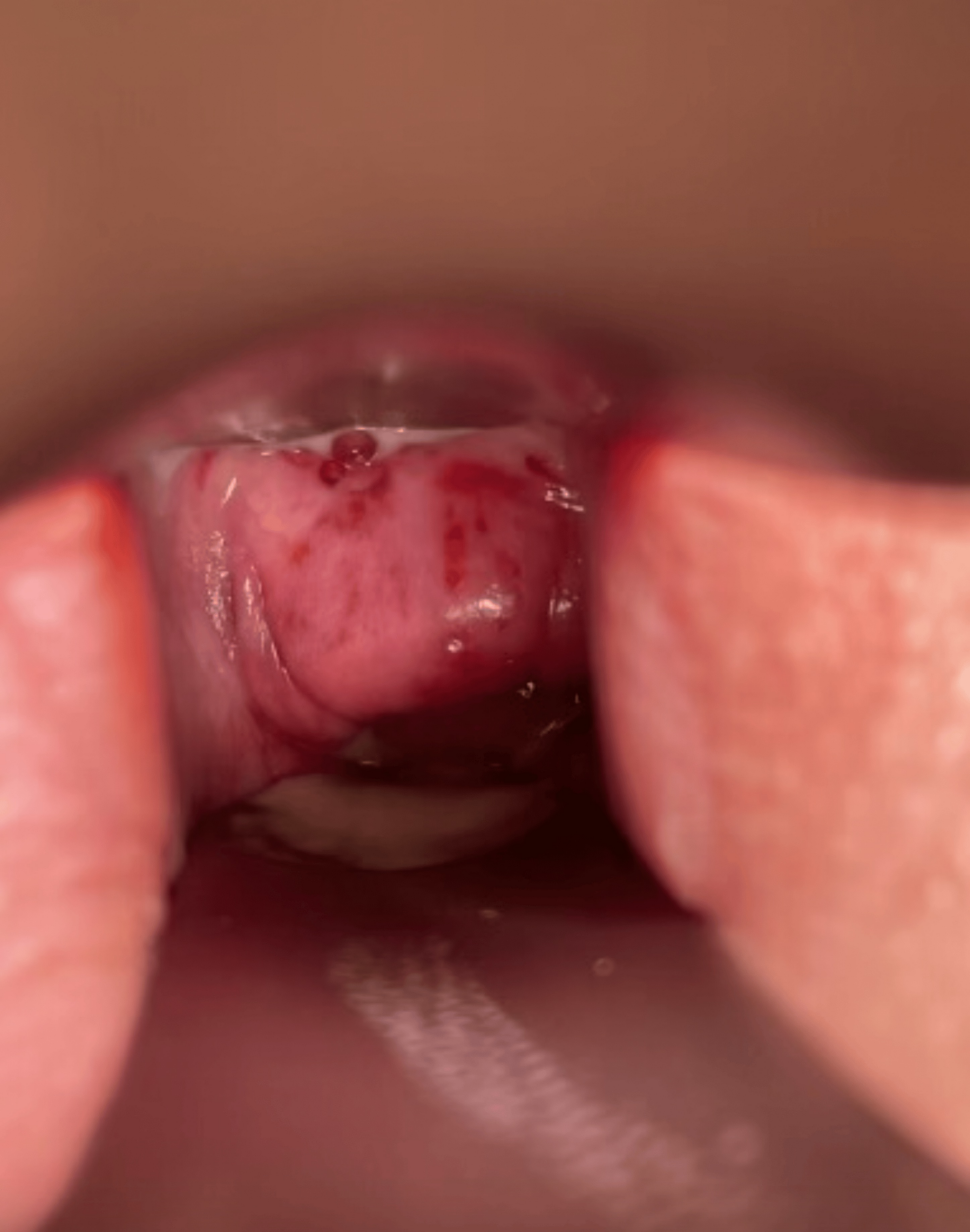
Cervix and vaginal walls of the patient 12 months after exposure to TCAA. Vaginal mucosa lacks normal mucosal folds and mucosal lubrication. TCAA: Trichloroacetic acid

Nearly one year following the incident, the patient reported that she continued treatment with a pelvic floor specialist. Her chemical burns on her buttocks and legs were still clearly demarcated in a spill/burn pattern and painful to the touch (Figure 1). On a speculum exam, irritation and scar tissue in her vagina and around her cervix were seen (Figure 2). At that time, she was still unable to tolerate intercourse or tampon insertion due to the pain. In response to asking how the patient was doing emotionally at this time, she wrote, "I’m heartbroken, despondent, and in constant fear that I will never be the same... All I wanted was a family, and all I got was scars for life."

Shortly thereafter, the patient underwent a frozen embryo transfer after standard estrogen and progesterone priming of her endometrium. She achieved a 15mm 'trilaminar' endometrial stripe during this treatment (anything above 7 mm to 8 mm is considered excellent/normal). She achieved a pregnancy in her first embryo transfer. This transfer resulted in the live birth of a female infant in May of 2024.

## Discussion

This case underscores an avoidable medical error with profound health consequences for the affected patient, namely second-degree burns to affected skin areas, desquamation of the vaginal mucosa, and dyspareunia. The well-documented protocols shown to avoid medical errors in the surgical or inpatient setting are not always observed in the outpatient setting. While beyond the scope of this case report, best practices include labeling all medications (even those in a syringe), double-checking labeling, and correctly passing on patient medicines to the next provider. Likewise, we underscore the 'five rights' of medication use: the right patient, the right drug, the right time, the right dose, and the right route.

This case also illustrates the importance of performing ‘time-outs’ prior to performing bedside procedures in the outpatient setting. Data has shown that the lack of standardization and timeouts during outpatient procedures contributes to medical errors [10]. Unfortunately, many of these safeguards are not performed in the context of overbooked appointments and understaffed clinics [10]. Healthcare systems must prioritize safety and training and must resource providers with the tools to prevent medical errors.

This case also illustrates the importance of multidisciplinary team management for complex medical presentations, especially in cases where overall knowledge and research on the topic are limited. Interdisciplinary care teams have been shown to have the potential to significantly impact patient and team experiences when caring for seriously ill patients [11]. A qualitative case study demonstrated that multidisciplinary collaboration resulted in better patient outcomes [12]. Our care team included obstetrics and gynecology, gynecologic oncology, toxicology, burns, general surgery, and behavioral health services. Together, the collaboration optimized patient care and addressed medical, surgical, and psychological elements of the patient’s condition. It was with this multi-disciplinary support and open discussion (providers, patient, family) that this patient was offered and improved with conservative management.

Finally, this case demonstrates the regenerative capacity of the endometrium. Despite harsh chemical exposure, there was recovery of the endometrium to respond to estrogen and progesterone as required to become receptive for embryo implantation. Local estrogen has been shown to promote vaginal epithelial regeneration and increase blood flow to the vagina following radiation treatments in cancer patients [13]. These studies, in addition to our case report, suggest that vaginal estrogen can be considered a safe and effective treatment for acid burns in the vagina. It is unclear whether the use of vaginal estrogen contributed to the patient’s ability to become pregnant following the incident; however, it was unlikely to have caused additional harm. What is clear is that endometrial stem cells are powerful allies in restoring fertility potential after chemical insult.

## Conclusions

We report a case of accidental TCAA instillation into the uterus, fallopian tubes, and pelvic cavity with retrograde spillage into the vagina, labia, perineum, thighs, and buttocks. Second-degree burns were treated with conservative management and resulted in healing over the long term, with persistent scarring and dyspareunia. Importantly, uterine function was unimpaired, and the patient carried a pregnancy to term. This outcome indicates a robust response of endometrial stem cells in restoring endometrial function.
